# Endoplasmic reticulum stress and therapeutic strategies in metabolic, neurodegenerative diseases and cancer

**DOI:** 10.1186/s10020-024-00808-9

**Published:** 2024-03-20

**Authors:** Siqi Yuan, Dan She, Shangming Jiang, Nan Deng, Jiayi Peng, Ling Ma

**Affiliations:** Department of Clinical Laboratory, Union Hospital, Tongji Medical College, Huazhong University of Science and Technology, 430022 Wuhan, China

**Keywords:** Endoplasmic reticulum stress, Signaling pathway, Cancer, Neurodegenerative diseases, Metabolic, Therapeutic strategies

## Abstract

The accumulation of unfolded or misfolded proteins within the endoplasmic reticulum (ER), due to genetic determinants and extrinsic environmental factors, leads to endoplasmic reticulum stress (ER stress). As ER stress ensues, the unfolded protein response (UPR), comprising three signaling pathways—inositol-requiring enzyme 1, protein kinase R-like endoplasmic reticulum kinase, and activating transcription factor 6 promptly activates to enhance the ER’s protein-folding capacity and restore ER homeostasis. However, prolonged ER stress levels propels the UPR towards cellular demise and the subsequent inflammatory cascade, contributing to the development of human diseases, including cancer, neurodegenerative disorders, and diabetes. Notably, increased expression of all three UPR signaling pathways has been observed in these pathologies, and reduction in signaling molecule expression correlates with decreased proliferation of disease-associated target cells. Consequently, therapeutic strategies targeting ER stress-related interventions have attracted significant research interest. In this review, we elucidate the critical role of ER stress in cancer, metabolic, and neurodegenerative diseases, offering novel therapeutic approaches for these conditions.

**Supplementary Information:**

The online version contains supplementary material available at 10.1186/s10020-024-00808-9.

## Introduction

Proteins are essential cellular components critical for maintaining and regulating biological functions. Deviations in protein expression, folding, function, or localization can significantly affect the biological functions of organisms(Jayaraj et al. 2020). The ER is the primary site for protein and lipid synthesis and calcium ion storage in eukaryotic cells, playing a vital role in regulating protein secretion and synthesis. Under normal conditions, only correctly folded proteins are secreted, while genetic mutations or stress conditions, such as hypoxia and oxidative stress, lead to the formation of misfolded proteins. The accumulation of misfolded proteins induces ER stress and triggers the UPR, which relieves ER stress by enhancing the ER’s protein folding capacity and degrading misfolded proteins. Persistent ER stress ultimately leads to cell death(Walter and Ron 2011).

Maintaining protein homeostasis is essential for the normal physiological activities of organisms. Chronic ER stress is increasingly recognized as a key factor in a growing number of human diseases(Dikic 2017). Research confirms that ER stress is activated in many human diseases, inducing cascades of responses that lead to malignant disease progression, and that inhibition of ER stress attenuates disease progression. Cancer and metabolic diseases are common and have high mortality rates, while neurodegenerative diseases have complex pathogenetic mechanisms and are currently incurable(Qian et al. 2024; Peng et al. 2023). This paper reviews the molecular mechanisms of ER stress in cancer, metabolic diseases, and neurodegenerative diseases, and summarizes potential therapeutic targets associated with ER stress to provide new strategies for treating these diseases.

### Signaling pathway involved in ER stress

The UPR engages with ER transmembrane proteins, including protein kinase R-like endoplasmic reticulum kinase (PERK), activating transcription factor 6 (ATF6), and inositol-requiring enzyme 1 (IRE1)-mediated signaling pathways within the ER(Walter and Ron 2011; Zhang and Kaufman 2008)Fig. 1). Bip, functioning as a molecular chaperone in the ER lumen, and known as glucose-regulated protein of 78 kDa (GRP78), remains inactive alongside ATF6, PERK, and IRE1, acting as UPR transmembrane pressure sensors in the homeostatic state of the intracellular environment(Schroder and Kaufman 2005). When unfolded proteins exceed normal levels and ER stress ensues, Bip is released from the UPR sensors, the activated UPR subsequently reduces protein translation and augments correct folding(Walter and Ron 2011; Ibrahim et al. 2019).

**Fig. 1:**
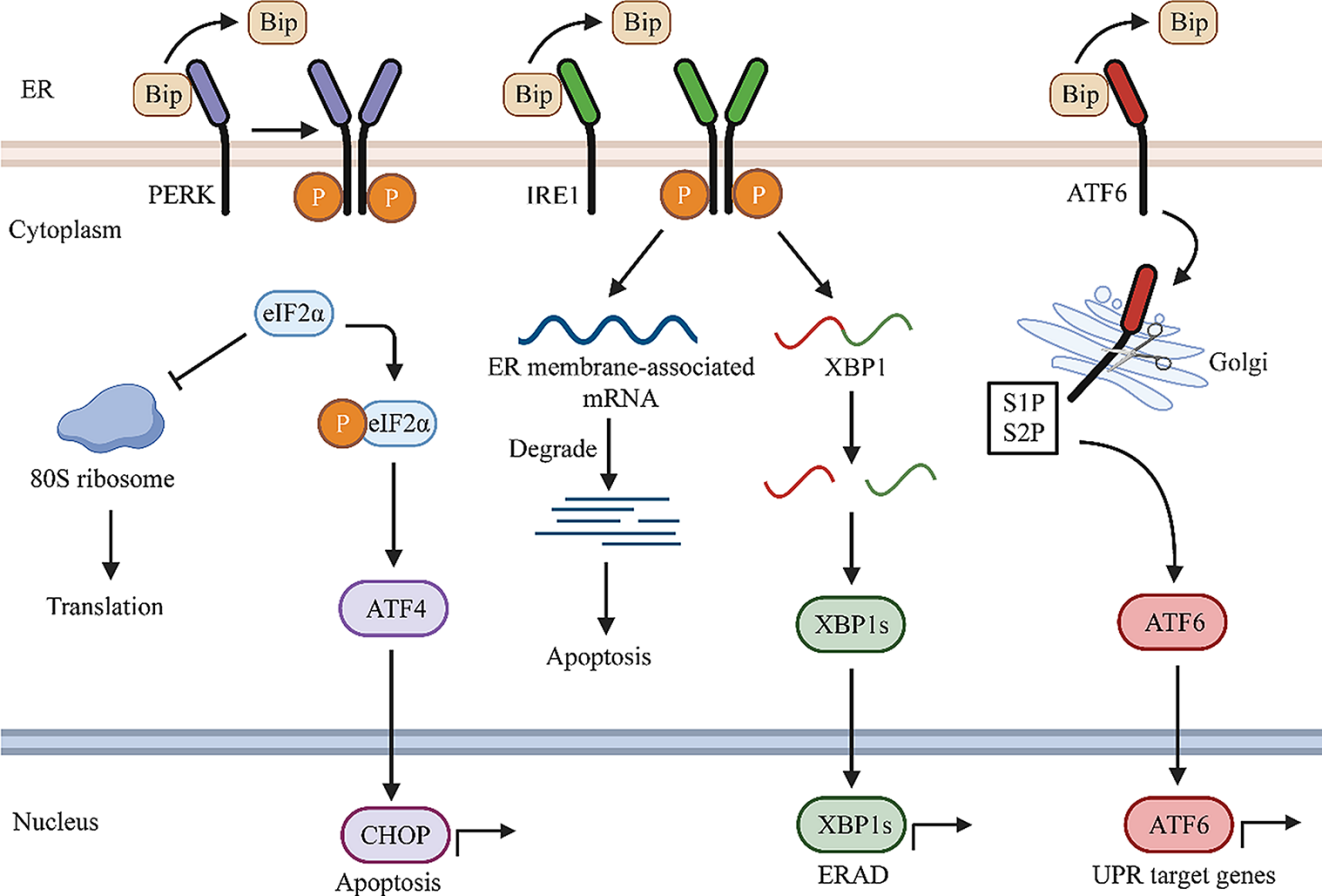
Summary of UPR signaling pathway. Under homeostatic conditions, PERK, IRE1, and ATF6 bind to immunoglobulin binding protein (Bip), inhibiting their activity. During ER stress, Bip dissociates, and PERK, IRE1, and ATF6 become activated. PERK dimerises and phosphorylates eIF2α, impeding the assembly of 80 S ribosomes, resulting in the inhibition of protein translation. Additionally, eIF2α induces the translation of activating transcription factor 4 (ATF4) and activates enhancer-binding protein C/EBP homologous protein (CHOP) expression, culminating in the induction of apoptosis. Activated IRE1 dimerises and triggers its endonuclease activity, leading to the degradation of mRNA in the ER and a consequent reduction in protein biosynthesis. Simultaneously, IRE1 cleaves the transcript encoding X box-binding protein 1 (XBP1), inducing endoplasmic reticulum-associated degradation (ERAD) gene expression, accelerating protein folding, and facilitating the degradation of misfolded proteins. ATF6 translocates to the Golgi, where it undergoes cleavage and activation by the S1 and S2 proteases, thereby activating the transcription of UPR target genes. (ER stress, endoplasmic reticulum stress; UPR, unfolded protein response; PERK, protein kinase R-like endoplasmic reticulum kinase; IRE1, inositol-requiring enzyme 1; ATF6, activating transcription factor 6; ATF4, activating transcription factor 4; Bip, immunoglobulin binding protein; eIF2α, eukaryotic initiation factor 2α; CHOP, enhancer-binding protein C/EBP homologous protein; XBP1, X box-binding protein 1; ERAD, endoplasmic reticulum-associated degradation.)

#### IRE1 signaling pathway

IRE1, a multifunctional protein exhibiting protein kinase and nucleic acid endonuclease activities, maintains an inactive monomeric conformation under unstressed conditions through binding with Bip in the luminal domains(Wiseman et al. 2022). In response to ER stress, IRE1 becomes activated through dissociation from Bip and binding to misfolded proteins(Hetz et al. 2020). This triggers IRE1 oligomerization, activation of its protein kinase activity, and subsequent autophosphorylation (Hetz et al. 2020; Gardner et al. 2013). Once activated, IRE1 executes signaling through three distinct mechanisms. Predominantly, phosphorylated IRE1 activates its nucleic acid endonuclease activity, binds to the mRNA of the precursor of XBP1, cleaves its intron, generating a new mRNA that translates into active XBP1(Lee et al. 2002). Functioning as a transcription factor, activated XBP1 binds to the promoters of endoplasmic reticulum stress response element (ERSE)-related genes, inducing the expression of foldase genes and ERAD genes. This accelerates protein folding and the degradation of misfolded proteins(Yoshida et al. 2003; Preissler and Ron 2019). XBP1 also exhibits nonspecific nucleic acid endonuclease activity, swiftly degrading ER membrane-associated mRNAs through modulation of the IRE1-dependent decay (RIDD) process, thereby reducing protein synthesis(Hollien et al. 2009; Hollien and Weissman 2006). This response alleviates stress by diminishing protein secretion.

#### PERK signaling pathway

PERK, a transmembrane protein comprising an ER stress-sensitive domain and a cytoplasmic kinase domain, parallels IRE1 in remaining inactive through binding with Bip. Under ER stress, Bip disengages from PERK, leading to PERK dimerization and autophosphorylation. Activated PERK then functions as a kinase, selectively phosphorylating the alpha subunit of eIF2α(Hetz et al. 2020). Phosphorylated eIF2α mitigates the accumulation of unfolded proteins by inhibiting the assembly of 80 S ribosomes, thereby suppressing protein translation and synthesis and reducing the influx of new proteins into the ER(Harding et al. 2003). Downstream of PERK, regulated genes include two transcription factors: ATF4 and CHOP. ATF4 mRNA expression levels rise significantly, accompanied by an increase in translation. ATF4 translocates to the cell nucleus, influencing downstream factors to promote cell survival by alleviating oxidative stress and fostering amino acid metabolism and cell survival(Harding et al. 2003). With persistent ER stress, ATF4 also activates the expression of proteins such as CCAAT enhancer-binding protein CHOP and DNA damage-inducible gene (growth-arrest and DNA damage-inducible gene 34 (GADD34), inducing apoptosis(Urra et al. 2013). This signaling pathway eases the burden by reducing the influx of proteins into the ER.

#### ATF6 signaling pathway

ATF6, a transmembrane protein characterized by an ER stress-sensing luminal domain and a cytoplasmic structural domain encoding the bZIP transcription factor, is integral to cellular response mechanisms(Wiseman et al. 2022). In the absence of ER stress, ATF6 resides in the ER in conjunction with Bip. Upon ER damage, Bip dissociates from the luminal domain, leading to an increased number of ATF6 monomers. These monomers subsequently traverse to the Golgi apparatus, where they undergo hydrolysis and processing facilitated by site 1 protease (S1P) and site 2 protease (S2P)(Haze et al. 1999). Activated ATF6 translocates to the nucleus, binds to ERSE-associated genes, and instigates their transcriptional activation (Adachi et al. 2008). The downstream target genes of ATF6 exhibit partial overlap with those induced by IRE1 and PERK, encompassing genes that facilitate protein folding, translocation, and synthesis (Adachi et al. 2008; MohanaSundaram et al. 2022). Additionally, ATF6 has the capability to form dimers with XBP1, activating ERAD-related genes. This pathway contributes to the promotion of protein folding, translation, and the mitigation of protein accumulation within the ER.

When ER stress becomes excessively intense or prolonged, exceeding the ER’s processing capacity, the IRE1, PERK, and ATF6 signaling pathways initiate an apoptotic cascade. This cascade activates downstream apoptosis-associated factors to eliminate damaged cells(Iurlaro and Munoz-Pinedo 2016). CHOP, a transcription factor, regulates the expression of various pro- and anti-apoptotic genes. It down-regulates anti-apoptotic proteins and up-regulates the pro-apoptotic protein Bim, leading to an increase in the expression of pro-apoptotic proteins Bax and Bak(Gu et al. 2017; Hu et al. 2018). ER stress results in significant increase in CHOP expression. Under non-stress conditions, Bax and Bak, located in the mitochondrial and ER membranes, bind to the anti-apoptotic protein Bcl-2, remaining in an inactive state. Simultaneously, Bim is inhibited through binding to cytoskeletal proteins. Activated IRE1α recruits the junction molecule TNF-receptor-associated factor 2 (TRAF2) and forms an IRE1-TRAF2-ASK1 complex with apoptosis signal-regulating kinase 1 (ASK1), subsequently activating c-Jun NH2-terminal kinase (JNK)(Urano et al. 2000). JNK phosphorylates Bcl-2 and CHOP inhibits Bcl-2 expression, together inhibiting the anti-apoptotic function of Bcl-2. Bax and Bak are activated upon dissociation from Bcl-2 and transmit apoptotic signals from the ER to the mitochondria to execute apoptotic functions(Szegezdi et al. 2006). Additionally, JNK phosphorylates Bim, which is released from the cytoskeleton and activates its pro-apoptotic functions. The caspase family plays crucial role in the death receptor and mitochondrial apoptosis. Caspase-12, located on the ER membrane is activated through the CHOP-ERO1α pathway and subsequently activates other caspase effector proteins, including caspase-9 and caspase-3, ultimately leading to apoptosis(Zhang et al. 2022a, b; Wu et al. 2020).

### The role of ER stress in metabolic, neurodegenerative diseases and cancer

**Fig. 2:**
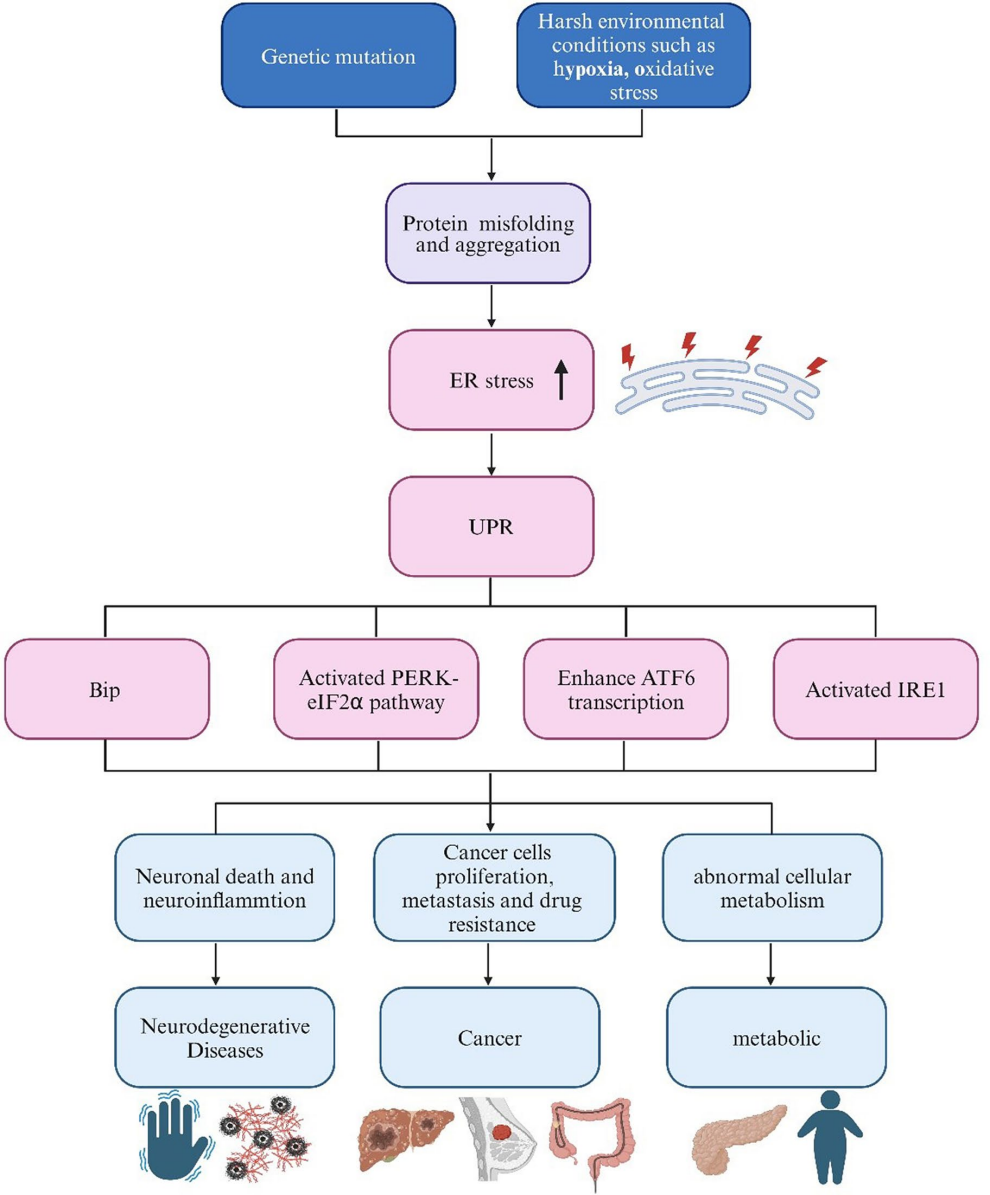
ER stress and human diseases triggered by misfolded protein aggregates. Environmental conditions, such as genetic mutations, hypoxia, and oxidative stress, disrupt ER homeostasis, leading to protein misfolding and accumulation. This, in turn, induces ER stress and expedites the build-up of disease-associated protein aggregates. The activation of the UPR signaling pathway serves to alleviate ER stress. However, when sustained ER stress surpasses the adaptive response capacity to manage protein misfolding, it can culminate in cell death, inflammation, and the proliferation of cancer cells, consequently contributing to disease progression. (ER stress, endoplasmic reticulum stress; UPR, unfolded protein response; PERK, protein kinase R-like endoplasmic reticulum kinase; IRE1, inositol-requiring enzyme 1; ATF6, activating transcription factor 6; Bip, immunoglobulin binding protein; eIF2α, eukaryotic initiation factor 2α.)

Altered ER protein homeostasis and aberrant UPR signaling are implicated in the development of various human diseases, including cancer, neurodegenerative disorders, metabolic diseases, and chronic inflammation(Hetz et al. 2020). The prospect of selectively manipulating UPR components through small molecules and genes represents a promising strategy for disease intervention (Hetz et al. 2020). The common denominator in these diverse diseases is the accumulation of misfolded proteins in the ER due to disruptions in protein folding caused by intracellular and/or extracellular conditions (Fig. 2). In the following sections, we will explore into several diseases intricately linked to ER stress, encompassing cancer, neurodegenerative diseases, and metabolic disorders.

### ER stress in metabolic disease

Metabolism refers to the series of chemical reactions that convert nutrients into energy necessary for organisms to grow, reproduce, and sustain life. The multicellular metabolic system of multicellular organisms facilitates the response to environmental changes such as nutrient availability, and temperature changes. Currently, metabolic diseases such as obesity, and diabetes are on the increase worldwide. The ER is a major organelle in lipid and carbohydrate metabolism. Thus, dysfunction of the ER is a key feature of metabolic disorders.

#### Diabetes

The ER, widely distributed in the cytoplasm of pancreatic β-cells, plays a crucial role in maintaining the internal environment required for insulin structure formation. Disturbances in ER protein homeostasis are closely associated with impaired pancreatic β-cell function in type 2 diabetes. Restoring ER homeostasis through small molecules emerges as a potential therapeutic strategy for type 2 diabetes. A recent study reported the efficacy of the chemical chaperone-like small molecule, KM04794, in alleviating ER stress. KM04794 demonstrated effectiveness in mitigating protein aggregation and cell death induced by insulin mutant proteins(Miyake et al. 2022).Persistent stimulation with N-methyl-D-aspartate (NMDA) has been reported to induce β-cell apoptosis and impact insulin secretion(Huang et al. 2017a, b). This effect is primarily attributed to NMDA treatment inducing ER stress in MIN6 β-cells, evidenced by increased expression of ATF4, CHOP, Bip, and XBP1s. Furthermore, blocking NMDA receptors (NMDARs) has been demonstrated to mitigate ER stress induced by high glucose concentrations in vitro and in vivo(Huang et al. 2019). β-cells lacking PERK exhibited abnormal morphology with ER expansion and insulinogenic retention. Diabetes associated with PERK deficiency is attributed to insufficient β-cell proliferation and defective insulin secretion. Overexpression of PERK accelerated diabetes progression in a mouse model, while PERK inhibition (PI) slowed its advancement(Gupta et al. 2010). Another study illustrated that low-dose PI treatment, inhibiting partial PERK phosphorylation, enhanced glucose-stimulated insulin secretion (GSIS) in mouse and human islets (Kim et al. 2019). Studies showing that ATF4 knockout mice suffer from severe diabetes characterized by hyperglycemia at a young age and a significantly shorter lifespan due to diabetic ketoacidosis. Additionally, ATF4 knockout mice exhibit pancreatic β-cell deficiency(Kitakaze et al. 2021).

Studies have shown that prolonged exposure to elevated glucose levels or the overexpression of IRE1α leads to the degradation of insulin mRNA in β-cells. Conversely, inhibition of IRE1α reduces insulin mRNA degradation. This phenomenon was further confirmed in a murine model, where pancreatic islets of IRE1α heterozygous mice exhibited higher insulin mRNA expression after chronic exposure to elevated glucose levels compared to their wild-type counterparts. These findings suggest that IRE1α expression contributes to insulin mRNA degradation during chronic high glucose-induced ER stress, thereby alleviating ER stress(Lipson et al. 2008). In mice with obesity or a high-fat diet, deletion of XBP1 in β-cells results in diabetes mellitus and exacerbates glucose intolerance by impairing insulin secretion(Lee et al. 2022). Type 1 diabetes, an autoimmune disease triggered by immune dysregulation and autoreactive T cells against pancreatic β-cells, induces β-cell apoptosis during disease progression. Inhibition of IRE1α RNase hyperactivity reduces β-cell apoptosis and reverses Type 1 diabetes in a non-obese diabetic (NOD) mouse model(Morita et al. 2017). Destruction of pancreatic β-cells due to immune-mediated causes leads to Type 1 diabetes. Knockdown of IRE1α induces β-cell dedifferentiation in NOD mice before the onset of pancreatic islet inflammation, resulting in reduced immune cell infiltration in the pancreatic islets and β-cell apoptosis. Moreover, the number of cytotoxic CD8 + T cells is reduced in the pancreas of IRE1α knockout mice(Lee et al. 2020).

#### Obesity

Obesity is a global health crisis that affects virtually all organ systems, caused by a complex interaction between genes and environmental factors, leading to disturbances in cellular metabolism and disruption of homeostasis (Ajoolabady et al. 2022). Obesity is characterized by chronic inflammation due to abnormal or excessive lipid accumulation in adipose tissue. A study found that high-fat diet-induced obesity induces ER stress and causes chronic inflammation in adipose tissue, whereas an inhibitor of ER stress alleviated metabolic disturbances and reduced the expression of inflammatory cytokines in high-fat diet (HFD) -obese mice, as well as reduced free cholesterol in adipose tissue(Chen et al. 2016). One of the main theoretical reasons for the development of obesity is the imbalance between M1 (pro-inflammatory) and M2 (anti-inflammatory) macrophage polarization. IRE1α was shown to be a key inducer in the regulation of M1-M2 macrophage polarization. In a mouse model, inhibition of IRE1α reversed HFD-induced white adipose tissue (WAT) M1-M2 imbalance and prevented HFD-induced obesity (Shan et al. 2017). Thus, inhibition of IRE1α may be an alternative for the therapy of obesity.

The Homeobox a5 (Hoxa5) gene, a developmental transcription factor, is highly expressed in adipose tissue. Hoxa5 attenuates ER stress by inhibiting the eIF2α/PERK signaling pathway in adipocytes and promotes M2 macrophage polarization, and which in turn attenuates obesity-induced chronic inflammation. This finding suggests that Hoxa5 may be a potential therapeutic target for obesity(Cao et al. 2019). Ghrelin, a gut hormone, circulates in acylated and deacylated forms. In obese rats, both forms of ghrelin downregulate the ER stress signaling molecules Bip, IRE1α, PERK, ATF6, ATF4, and CHOP, and inhibit hepatocyte steatosis and inflammation(Ezquerro et al. 2020).

It has been observed that obesity-induced ER stress in adipose tissue of mice is associated with reduced levels of adiponectin. Adiponectin, a peptide hormone produced by adipose tissue, promotes energy expenditure to suppress obesity. In mouse 3T3-L1 adipocytes, induction of ER stress promotes adiponectin degradation. Conversely, inhibition of ER stress increases lipocalin levels and attenuated high-fat diet-induced reduction of adiponectin in mice(Zhou and Liu 2010). In summary, obesity leads to abnormal cellular metabolism and disturbed ER homeostasis, causing ER stress, which in turn aggravates obesity by inhibiting energy expenditure. Thus, alleviating ER stress may be a therapeutic target to improve obesity.

Large fluctuations in nutrient supply lead to stress for nutrient synthesis, and the ER, an important organelle for maintaining metabolic stability, adapts to the altered environment by activating the UPR. However, when the adaptation of the ER fails, it leads to metabolic abnormalities, organelle dysfunction, and insulin resistance. Preclinical cellular animal models have also progressed, such as small molecule targets of ER stress in β-cells that attenuate metabolic dysfunction. Thus, attenuating ER stress is a promising therapeutic strategy for the treatment of metabolic disorders.

### ER stress in neurodegenerative diseases

Most neurodegenerative diseases are pathologically characterized by the abnormal aggregation of misfolded proteins and the formation of neuronal inclusion bodies. The toxic accumulation of protein aggregates leads to progressive neuronal damage and neurodegeneration. Numerous studies have identified early manifestations of ER stress in the symptomatology of many neurodegenerative diseases, implying a potential contribution of ER stress to the onset of neurodegeneration(Kim et al. 2022). Common neurodegenerative diseases include Parkinson’s, Huntington’s, Alzheimer’s disease, amyotrophic lateral sclerosis, and prion disease, among others. With the ageing population, Parkinson’s and Alzheimer’s diseases stand as the top two neurodegenerative diseases, posing significant threats to the life and health of the elderly(Xiang et al. 2023). Huntington’s disease, a typical autosomal dominant neurodegenerative disease, results from a gene mutation. The pathological changes at the onset of neurodegenerative diseases are irreversible, and due to the multiplicity of pathogenic factors, targeting one or two pathways cannot substantially mitigate the overall dysfunction and loss of neurons. As research on neurodegenerative diseases intensifies, leveraging the advantages of multi-pathway and multi-targeted treatments holds the potential to improve symptoms and regulate brain function in patients with neurodegenerative diseases.

#### Alzheimer’s disease

Alzheimer’s disease (AD) is a devastating neurodegenerative disease that is the major cause of dementia and is characterized by a progressive decline in cognitive functioning(Ghemrawi and Khair 2020). The pathology of AD is characterized by the accumulation of amyloid β (Aβ) and phosphorylated tau (P-tau) in the patient’s brain, with tau being a brain-specific protein that leaks from mesenchyme to plasma in AD (Mattsson et al. 2016; Scheltens et al. 2021). Tau accumulation disrupts ER protein homeostasis and activates the UPR by disrupting ERAD pathways, leading to ER stress(Abisambra et al. 2013).

Bip serves as a crucial marker of ER stress, with increased expression observed in the AD mouse model, indicating the presence of ER stress in the in vivo model of AD. The expression of IRE1α, p-p38, and p-NF-κB is elevated in AD mice, suggesting the activation of NF-κB and p38 inflammatory pathways through the IRE1α axis, leading to neuroinflammation(Dsouza et al. 2022). Mutant amyloid precursor protein (APP) promotes the accumulation of toxic Aβ oligomers, inducing ER stress in the ER, resulting in the upregulation of GRP78 and p-eIF2α expression (Nishitsuji et al. 2009; Soejima et al. 2013). Deletion of IRE1 in a mouse model of AD reduces APP expression, and in vitro experiments demonstrate that inhibiting IRE1 signaling disrupts APP homeostasis, causing APP retention in the ER and triggering ER stress(Duran-Aniotz et al. 2017). Paradoxically, overexpression of XBP1, a downstream transcription factor of IRE1, in the nervous system of AD mice reduces amyloid deposition and safeguards synaptic and cognitive functions(Duran-Aniotz et al. 2023). This suggests that XBP1 plays an important role in memory and cognitive functions(Cisse et al. 2017). Additionally, in an in vitro study of SK-N-SH human neuroblastoma cells, Aβ preferentially activates the PERK-eIF2α pathway, and silencing PERK leads to cell death, which is mitigated by Salubrinal, a selective activator of eIF2α(Bobrovnikova-Marjon et al. 2010). ATF6, a crucial ER stress sensor, is reduced in the AD mouse model, and in the cellular model, ATF6 downregulation decreases APP expression (Zhang et al. 2022a, b). Moreover, knockdown of ATF6 disrupts spatial memory retention in AD mice (Du et al. 2020). The above studies indicate that ER stress is mostly malignantly associated with AD, and that inhibition of ER stress in cellular and animal models attenuates AD symptoms and may provide some molecular therapeutic targets that are effective in AD.

#### Parkinson’s disease

One of the main features of Parkinson’s disease (PD) is the aggregation and accumulation of misfolded proto fibrillar alpha-synuclein (α-syn) in neurons, forming structures known as Lewy bodies (LBs)(Kim et al. 2022, Mercado et al. 2016). Aggregated α-syn induces ER fragmentation, which reduces ER’s ability to fold proteins and process misfolded proteins, leading to ER stress(Stojkovska et al. 2022). Studies have demonstrated that α-syn hinders the protective UPR to ER-Golgi vesicle transport by ATF6, leading to sustained chronic ER stress and subsequent cell death(Credle et al. 2015). Overexpression of Bip inhibits apoptosis and enhances the survival of nigral dopamine (DA) neurons in a rat model of PD with elevated α-syn, concurrently reducing the neurotoxicity associated with α-syn (Gorbatyuk et al. 2012). Additionally, the pathological features of PD encompass dopaminergic neuronal degeneration(Manaa et al. 2021). Expression of adenoviral XBP1s vectors significantly inhibits dopamine neuron degeneration in a 1-methyl-4-phenyl-1,2,3,6-tetrahydropyridine (MPTP)-induced PD mouse model, while the exogenous expression of active XBP1s proteins protects against 1-methyl-4-phenylpyridinium (MPP+)-induced cell death (Sado et al. 2009). Furthermore, the expression of GRP78, total and phosphorylated PERK, eIF2α, and CHOP is upregulated in a rat model of PD and autopsy tissues from PD patients(Motawi et al. 2022; Mercado et al. 2018). Thus, current therapeutic strategies for PD aim to enhance the ER’s ability to process folded proteins, stabilize the internal environment’s homeostasis, and reduce the negative effects of persistent and chronic ER stress.

#### Huntington’s disease

Huntington’s disease (HD) is an inherited disorder caused by mutations in the gene encoding the Huntington protein (Htt) and is characterized by movement disorders, cognitive decline, and psychiatric disorders(Sakahira et al. 2002). The abnormally prolonged polyglutamine (polyQ) in the synthesized Huntington protein (Htt) forms large molecular clusters that accumulate in the brain, causing neurodegeneration(Finkbeiner 2011). Htt mutations lead to the production of misfolded Htt protein, and the misfolded proteins interact and aggregate intracellularly, inducing ER stress and cytotoxicity(Shacham et al. 2019). It has been shown that inhibition of ERAD leads to the accumulation of unfolded proteins in the ER, inducing ER stress(Leitman et al. 2013). ER stress is implicated in HD, with the expansion of the ER being a notable feature. Striatal cells established in HdhQ111 knock-in embryos exhibited significant alterations in ER morphology compared to the wild type, indicating ER expansion(Trettel et al. 2000). HD is characterized by the aggregation of mutant Huntington (mHtt) protein. In a study, the expression of mHtt exon 1 in mouse cells activated IRE1, leading to the degradation of biogenesis of lysosome-related organelles complex 1 subunit 1 (Blos1). The override of Blos1 degradation resulted in the excessive accumulation of mHtt aggregates(Bae et al. 2022). Further investigations demonstrated that ER stress injury increased mHtt aggregation through IRE1 activation in neuronal cells. This study also revealed that the kinase activity of IRE1 stimulates mHtt aggregation, exacerbating neuronal cell death(Lee et al. 2012). At the cellular level, IRE1 is activated in the striatum of mutant Htt transgenic (BACHD) mice(Hyrskyluoto et al. 2014). Emotional and cognitive deficits in HD patients arise from hippocampal dysfunction. ER stress activation was observed in the early stages of HD in the hippocampus of R6/1 mice (HD models), evidenced by increased expression of GRP78 and CHOP. Spatial and recognition memory restoration occurred in R6/1 mice after inactivating PERK(Espina et al. 2023). HD is characterized by the aggregation of toxic Huntington proteins. In brain tissues from striatal cell lines expressing pathogenic Huntington proteins and from HD mouse models, the phosphorylation level of eIF2α was elevated by increasing PERK activity, inducing ER stress. Inhibition of PERK significantly reduced Htt protein toxicity(Leitman et al. 2014). The activation of the PERK pathway, usually associated with the inhibition of protein translation and synthesis after eIF2α is phosphorylated by PERK, was shown to improve HD symptoms in cells (STHdhQ111/111) and mice (R6/2) when directly activated by the PERK activator MK-28. Additionally, MK-28 inhibited ER stress-induced apoptosis in vitro(Ganz et al. 2020). ER stress leads to the cleavage of ATF6α by Golgi enzymes during transport, releasing N-terminal ATF6α (Nt-ATF6α). In HD animal models and brains of HD patients, the ATF6α pathway is impaired, evidenced by a reduction in Nt-ATF6α and the accumulation of ATF6α. This impairment is accompanied by a decrease in Ras-homologue enriched in the brain (Rheb), linked to late neuronal death(Fernandez-Fernandez et al. 2011). Disruption of Ca^2+^ homeostasis can also induce ER stress. Subsequent studies revealed reduced expression of downstream regulatory element antagonist modulator (DREAM), a Ca^2+^-dependent transcriptional repressor, in an HD mouse model. The reduced expression of DREAM is considered a neuroprotective response, associated with the interaction between DREAM and ATF6. Blocking this interaction enhances ATF6 transcription, promoting the early survival function of striatal neurons(Naranjo et al. 2016). It is evident that all three signaling pathways of the UPR are associated with HD toxicity, and maintaining protein homeostasis is essential for preventing mHtt-related toxicity. Thus, modulation of the UPR pathway using specific inhibitors or activators may be an effective therapeutic approach.

Several studies, utilizing mouse models and in vitro cellular experiments, have consistently demonstrated that in neurodegenerative diseases, misfolded proteins aggregate and are deposited either intracellularly or extracellularly. This phenomenon disrupts the intracellular environment’s homeostasis, triggering ER stress. Prolonged ER stress is associated with neuronal cell death. The demise of neural cells compromises the regulation of the nervous system, and resultant neurological abnormalities likely exert negative effects on other physiological processes, including the development of cancer.

Neurodegenerative diseases are irreversible. and the exact pathogenesis is not fully understood. A common factor is that environmental and genetic factors contribute to genetic mutations, and different genetic mutations lead to the accumulation of different misfolded proteins in the ER, inducing ER stress, which in turn, results in neuronal death and neuroinflammation. Although it is not possible to completely inhibit the progression of the disease, studies have demonstrated that inhibiting ER stress alleviates symptoms and progression. Moreover, studies have shown that ER stress inhibitors could reduce drug toxicity and drug dosage, providing various ideas for the treatment of neurodegenerative diseases.

### ER stress in cancer

A defining characteristic of cancer cells is their propensity to metastasize, disseminating from local tissues to distant sites(Zhang et al. 2023). Environmental challenges in these tissues, such as hypoxia, hypoglycemia, growth factor deficiencies, lactic acidosis, oxidative stress, and amino acid starvation, disrupt the precise folding of proteins within the ER(Oakes 2020). Concurrently, cancer cells exhibit distinctive features, including aneuploidy, heightened metabolic demands, and the ability to proliferate incessantly. These attributes necessitate elevated levels of ER homeostasis and secretory mechanisms, intensifying stress on the cellular secretory pathway(Dejeans et al. 2014). Collectively, these conditions foster the accumulation of misfolded proteins in the ER, inducing ER stress and activating the UPR. Numerous studies have underscored the contribution of all three UPR signaling pathways to tumor growth(Urra et al. 2016; Lee 2007).

PERK orchestrates tumorigenesis through various mechanisms. Hypoxia, a hallmark of tumor cells and a key driver of malignancy and drug resistance(Yang et al. 2022), is counteracted by the PERK/eIF2α signaling pathway, which stimulates cysteine uptake, glutathione synthesis, and controls oxidative stress to safeguard hypoxia-tolerant cells(Rouschop et al. 2013). Additionally, epithelial-to-mesenchymal transition (EMT), a pivotal process in cancer leading to metastasis and drug resistance, necessitates PERK for the proliferation of EMT gene-expressing cells (Feng et al. 2014). Human lymphomas exhibit significantly elevated ER stress activation compared to normal tissues. The PERK/eIF2α/ATF4 arm, activated by c-Myc and n-Myc, enhances tumor cell survival through the induction of cytoprotective autophagy. Notably, inhibition of PERK proves efficacious in malignant tumors dependent on c-Myc overexpression(Hart et al. 2012). ATF6α plays a crucial role in the survival of dormant squamous carcinoma cells. Knockdown of ATF6α induces apoptosis in dormant cancer cells, prolonging the period of cell dormancy and delaying the onset of recurrent cancer cell growth(Li et al. 2022). Recent findings link ATF6 overexpression to the development of drug resistance in high-grade serous ovarian cancer (McMellen et al. 2023). In a human glioma model, inhibition of IRE1α led to downregulation of pro-angiogenic factors, and in a mouse model, reduced IRE1α expression resulted in diminished tumor growth(Auf et al. 2010). Overexpression of Myc induces ER stress through C-Myc- and N-Myc-dependent mechanisms, and Myc-overexpressing cells depend on the IRE1α/XBP1 signaling pathway for survival both in vitro and in vivo (Xie et al. 2018).

#### Hepatocellular carcinoma

Hepatocellular carcinoma (HCC) is the most common type of primary liver cancer and ranks among the most prevalent cancers globally. The liver, primarily functioning in metabolism, is severely affected in its metabolic function by the malignant transformation of hepatocytes. Risk factors for HCC include metabolic disorders such as obesity and type 2 diabetes, as well as B or C virus infection. HCC is characterized by abnormal metabolism and aberrant activation of ER stress(Luna-Marco et al. 2023).

Examination of the Human Protein Atlas has identified 44 UPR-related proteins as unfavorable prognostic markers for HCC. Notably, the expression of ATF4 and CHOP increased significantly as HCC progressed to stages 2 and 3. Concurrently, protein expression of the eIF2α-ATF4-CHOP pathway was upregulated in tumor tissues(Pavlovic and Heindryckx 2021). During HCC progression, activation of hepatic stellate cells promotes tumor cell proliferation and migration. A study found that HCC cells activate IRE1α in hepatic stellate cells, which promotes their activation, whereas inhibition of IRE1α prevents their activation and thus inhibits tumor cell proliferation and migration(Pavlovic et al. 2020). In HCC tissues, the mRNA expression of Bip and ATF6 increased and XBP1 was spliced and activated(Shuda et al. 2003). In the HCC mouse model, the PERK pathway was activated during tumor progression, CHOP increased, and morphologically, the electron microscopy results showed ER expansion in hepatocellular carcinoma cells. While a PERK inhibitor significantly reduced the tumor burden in the mouse model(Vandewynckel et al. 2015). One of the reasons HCC remains intractable to therapy is the high level of intrinsic drug resistance, and three signaling pathways of the ER stress signaling pathway have been shown to induce chemoresistance(Khaled et al. 2022).

Targeting ER stress signaling pathways and related molecules plays a crucial role in anti-HCC, therefore, all these markers can be considered as potential therapeutic targets for HCC.

#### Breast cancer

Breast cancer (BC) is the most common cancer among women, with its incidence increasing yearly. It is widely recognized that aberrant activation of ER stress and upregulation of ER stress components are involved in the progression of BC cells and the development of drug resistance(Sisinni et al. 2019).

In a study, immunohistochemical staining of tissues from 395 human BCs revealed that Bip and XBP-1 were expressed in 76% and 90% of breast cancers, respectively(Scriven et al. 2009). Bip inhibits apoptosis induced by estrogen starvation in human breast cancer cells by binding to proapoptotic protein BCL-2 interacting killer (Bik), thus developing resistance to hormonal therapies that block estrogen synthesis(Fu et al. 2007). The PERK/eIF2α axis promotes tumor growth by preserving redox homeostasis in an animal model of breast cancer(Bobrovnikova-Marjon et al. 2010). PERK conveys stress signals to the NF-κB pathway to regulate estrogen-induced apoptosis in breast cancer cells(Fan and Jordan 2018). In estrogen receptor-positive (ERα+) BC cells, estrogen acts through ERα to rapidly activate ER stress, leading to increased Bip production and benefiting cancer cell proliferation(Andruska et al. 2015). In addition to the PERK signaling pathway, the IRE1α-XBP1 signaling plays an important role in the development of ERα + BC. ERα is expressed in up to 70% of breast cancers and patients are usually treated with endocrine therapy. However, resistance often develops with time, leading to aggression of the disease. XBP1 is significantly over-expressed in ERα + BC, which is related to anti-estrogen resistance(Barua et al. 2020). Moreover, Myc in breast cancer enhances IRE1α transcriptional activity by forming a complex with XBP1(Zhao et al. 2018). In conclusion, the ER stress signaling pathway is involved in and contributes to the occurrence and progression of BC, leading to drug resistance, and inhibition of ER stress contributes to restoring drug sensitivity.

#### Colorectal cancer

Colorectal cancer (CRC) is the third most prevalent malignant tumor globally and has a high mortality rate, currently ranking as the fourth leading cause of death worldwide(Li et al. 2021; Zhou et al. 2023). In CRC, tumor cells are usually highly proliferative and metabolically active, which may lead to abnormal protein synthesis and ER stress. ER stress may influence CRC progression by regulating tumor cell survival, proliferation, and drug resistance(Shi et al. 2024). Additionally, hypoxia, a common feature of the tumor microenvironment (TME), disrupts the homeostasis of the ER’s internal environment and induces stress in the ER lumen.

Activation of the eIF2α-ATF4 axis and increased phosphorylated eIF2α both contribute to chemoresistance in CRC(Guo et al. 2021). Expression of XBP1s promotes the proliferation of colon cancer cells (Liu et al. 2023). In a risk model of ER stress-related genes (ERSRGs) for prognostic assessment and treatment of CRC patients, the high-risk group was shown to be more resistant to the chemotherapeutic agent 5-Fluorouracil (5-FU), and risk scores were positively correlated with 5-FU resistance(Geng et al. 2023). ATF6 expression is detected in colorectal cancer but is absent in normal colonic mucosa. It serves as a marker for pre-cancerous atypical lesions in CRC associated with non-ulcerative colitis and ulcerative colitis(Hanaoka et al. 2018). Elevated expression of the secreted protein Gremlin-1 (GREM1) correlates positively with poor CRC prognosis, and GREM1 upregulates ATF6 expression(Pavlovic et al. 2020).

When ER stress is activated in the organism, the UPR triggered initially serves to restore the homeostasis of the internal environment of the ER, which in turn supports cell survival. However, if ER stress persists, the cell shifts from an adaptive response to an induced death response, suggesting that there is a certain potential threshold of ER stress before apoptosis. Consequently, mastering the intensity and duration of ER stress to restore ER stability is a feasible new target for antitumor therapy.

## Therapeutic strategies

In the majority of the aforementioned diseases, there is an augmented expression of UPR signaling molecules in target cells. Knockdown or inhibition of these signaling molecules has been shown to impede disease progression, presenting them as potential therapeutic targets. This review succinctly outlines a selection of UPR modulators that have exhibited efficacy in clinical models of cancer, neurodegenerative diseases, and diabetes mellitus (Table 1).

**Table 1:** Potential small inhibitor targeting ER stress in preclinical and clinical. (ER stress, endoplasmic reticulum stress; PERK, protein kinase R-like endoplasmic reticulum kinase; IRE1α, inositol-requiring enzyme 1α; Bip, immunoglobulin binding protein.)

Molecule	Mechanism	Indication	Experimental study	Refs
HA15	Bip inhibitor	Osteosarcoma	In vivo	92, 115
OSU-03012	Bip inhibitor	Osteosarcoma	In vitro	92
IKM5	Bip inhibitor	Breast cancer	In vivo	116
KP1339	Bip inhibitor	Colon cancer	Phase I	119
STF-083010	IRE1α inhibitor	Multiple myeloma	In vitro	120
B-I09	Selective IRE1α RNase inhibitor	Chronic lymphocytic leukemia	In vivo	121
4µ8C	IRE1α-specific inhibitor	Colon cancer	In vitro	122
Compound 18	IRE1α kinase inhibitor	Tumor	In vitro	123–125
SCD1	IRE1α RNase activity inhibitor	Burkitt’s lymphoma	In vitro	93
MKC8866	IRE1α RNase inhibitor	Glioblastoma multiforme	In vivo	126
GSK2606414	PERK inhibitor	Diabetes, neurodegenerative diseases	In vivo	127–129
GSK2656157	PERK inhibitor	Diabetes, colon cancer	In vivo	128, 130
Small molecule 42,215	PERK small molecule inhibitor	Colorectal cancer	In vitro	131

### Bip inhibitors

Bip inhibitors, including HA15 and OSU-03012, demonstrated heightened toxicity towards canine osteosarcoma cells compared to osteoblastic progenitor cells from normal bone(Mattos et al. 2022). HA15 exhibited the ability to induce cancer cell death both in vivo and in vitro and impede melanoma development in mice(Cerezo et al. 2016). IKM5, another Bip inhibitor, hindered breast tumor growth, complementing the inhibitory effects of Doxorubicin in the early therapeutic phase of breast cancer (Nayak et al. 2019). Additionally, the Bip inhibitor KP1339 showcased promising anticancer activity in phase I clinical trials (Schoenhacker-Alte et al. 2017; Burris et al. 2016). In a colon cancer model, KP1339 induced immunogenic cell death (ICD), leading to a sustained immune response against the tumor (Wernitznig et al. 2019). Given the upregulation of Bip expression in various diseases, inhibiting Bip expression emerges as a therapeutic strategy, demonstrating potential value in further research.

### IRE1α inhibitors

A study unveiled that the small molecule inhibitor of IRE1, STF-083010, exhibited significant antimyeloma activity in simulated human multiple myeloma (MM) xenografts, inhibiting IRE1 endonuclease activity both in vitro and in vivo(Papandreou et al. 2011). The selective IRE1 RNase inhibitor B-I09 hindered the transmembrane receptor for IRE1, showing efficacy in inhibiting leukemia progression without systemic toxicity in a mouse model of chronic lymphocytic leukemia (CLL)(Tang et al. 2014). The IRE1α-specific inhibitor 4µ8C inhibited β-catenin production, a key factor in colon tumorigenesis, thereby suppressing the proliferation of colon cancer cells (Li et al. 2017). Additionally, the IRE1α kinase inhibitor Compound 18 demonstrated inhibition of tumor growth (Moore et al. 2019; Harnoss et al. 2020; Chen and Cubillos-Ruiz 2021). The IRE1α RNase activity inhibitor stearoyl-CoA-desaturase 1 (SCD1) attenuated cytotoxicity induced by standard chemotherapeutic agents in Burkitt’s lymphoma with c-Myc overexpression (Xie et al. 2018). Treatment with the IRE1α RNase inhibitor MKC8866 significantly improved survival in the Glioblastoma multiforme (GBM) mouse model (Reste et al. 2020). In conclusion, these preclinical studies suggest that pharmacological inhibitors of IRE1α may be beneficial in delaying disease progression and improving clinical endpoints.

### PERK inhibitors

Common PERK inhibitors, GSK2606414 and GSK2656157, have shown notable effects in various studies(Chen and Cubillos-Ruiz 2021). In vitro, low-dose GSK2606414 treatment increased glucose-stimulated insulin secretion (GSIS) levels and pancreatic islet insulin content in mouse and human islets, while in vivo treatment with GSK2656157 enhanced GSIS and alleviated hyperglycemia in an insulin-deficient mouse model(Kim et al. 2018, 2019). Treatment of a mouse model of tau protein mutation-mediated dementia with GSK2606414 restored correct protein synthesis, prevented further neuronal loss, slowed brain atrophy, and suppressed clinical symptoms(Radford et al. 2015). GSK2606414 also attenuated pathological damage to hypothalamic neurons(Yi et al. 2019). In a mouse model, the PERK inhibitor GSK2656157 synergistically inhibited the growth of colon cancer cells with 5-FU, a commonly used first-line chemotherapeutic agent for advanced colon cancer patients. Furthermore, GSK2656157 demonstrated effectiveness in sensitizing CRC cells with enhanced resistance to 5-FU treatment (Shi et al. 2019). A recent study found that the PERK small molecule inhibitor 42,215 exhibited excellent antitumor efficacy, inducing apoptosis and G2/M cell cycle arrest in a dose- and time-dependent manner in HT-29 human colon adenocarcinoma(Rozpedek et al. 2020). Therefore, PERK inhibitors show promise in antitumor efficacy, improving resistance to drug therapy, and reducing clinical symptoms.

### Clinical trials

Currently, there are clinical trials of drugs used as ER stress inhibitors for the treatment of human diseases (Table 2). Tauroursodeoxycholic acid (TUDCA) is a hydrophilic bile acid usually produced endogenously in the human liver from taurine conjugated with ursodeoxycholic acid (UDCA). TUDCA has been used in the clinical treatment of hepatobiliary disorders and is a well-recognized inhibitor of ER stress(Yuan et al. 2021). Recent clinical trials have used TUDCA as an ER stress inhibitor in the treatment of diabetes, cardiovascular disease, and human hypertension. UDCA is also a clinically approved drug for the dissolution of cholesterol gallstones, primary biliary cholangitis, and other hepatobiliary diseases(Goossens and Bailly 2019). A clinical trial suggests that UDCA, a potent ER stress inhibitor, protects against peritoneal fibrosis in peritoneal dialysis. Furthermore, more clinical trials confirm that ER stress and human disease are correlated.

**Table 2:** ER stress inhibitor in clinical trials. (TUDCA, Tauroursodeoxycholic acid; UDCA, Ursodeoxycholic acid)

Drug	Mechanism	Disease	Clinical trial	NCT number
TUDCA	ER stress inhibitor	Type 1 Diabetes	Phase 2	02218619
TUDCA	ER stress inhibitor	Human Hypertension	Phase 2	06025630
TUDCA	ER stress inhibitor	cardiovascular disease	Early Phase 1	04001647
UDCA	ER stress inhibitor	Peritoneal fibrosis	Phase 4	02338635

The therapeutic targets discussed above are all small molecule inhibitors of ER stress, and most of them are still in preclinical studies due to their toxicity, safety, and metabolic processes in vivo not being fully defined yet. These in vivo or in vitro trials only provide some new therapeutic strategies and perspectives, and a great number of experimental studies are needed if they are to enter clinical use.

## Conclusions and future prospects

The accumulation of misfolded proteins within the ER induces ER stress, triggering the UPR with the aim of reinstating protein homeostasis. The UPR initiates by enhancing ER protein processing capacity and reducing the ER load to re-establish ER homeostasis. However, with persistent ER stress, the UPR generates inflammatory and death signals, ultimately culminating in cell death. Chronic ER stress plays a pivotal pathological role in numerous diseases. In recent years, ER stress has been widely discussed as a potential therapeutic target for human diseases. This article reviews the molecular mechanisms of ER stress in metabolic, neurodegenerative diseases and cancer, in addition to the potential ER stress-related therapeutic targets. Most of the therapeutic targets that have been studied and reported are from experimental studies in cellular or animal models, and few have been used in human clinical trials, which at least provide research directions for new therapeutic strategies for ER-related diseases despite these limitations. The insights gained are anticipated to pave the way for exploring novel therapeutic strategies for ER stress-related diseases.

### Electronic supplementary material

Below is the link to the electronic supplementary material.

Supplementary Material 1

Supplementary Material 2

## Data Availability

Not applicable.
