# Supplementary material for: Endoplasmic reticulum stress and therapeutic strategies in metabolic, neurodegenerative diseases and cancer
Source: Mol Med. 2024 Mar 20;30:40. doi: 10.1186/s10020-024-00808-9 (PMC10956371; doi:10.1186/s10020-024-00808-9)
Supplement: Supplementary file 2 — Supplementary Material 2 [file 10020_2024_808_MOESM2_ESM.pdf]

• MedSci •

Editing By Professional Editors

CERTIFICATE OF ENGLISH EDITING

This document certifies that the manuscript entitled "Endoplasmic reticulum stress and therapeutic strategies in metabolic, neurodegenerative diseases and cancer" was proofread and edited for proper English language, grammar, punctuation, spelling, and overall style by one or more of the qualified scientific editors at MedSci, all of whom are native English speakers. Neither the research content nor the authors' intentions were altered in any way during the editing process.

Documents receiving this certification should be English-ready for publication; however, the author can accept or reject our suggestions and changes. To see the final MedSci edited version, please visit our verification page. If you have any questions or concerns about this document or certification, please contact us at editing@medsci.cn.

Corresponding Author:Ling Ma

DATE:2024-02-29

SIGNATURE: MedSci

CODE:0229-4CD8-3337-7490-C4AC

This certificate may be verified at

https://editing.medscihealthcare.com/dist/medsci-order/#/verify
