# Supplementary material for: Endoplasmic reticulum stress and therapeutic strategies in metabolic, neurodegenerative diseases and cancer
Source: Mol Med. 2024 Mar 20;30:40. doi: 10.1186/s10020-024-00808-9 (PMC10956371; doi:10.1186/s10020-024-00808-9)
Supplement: Supplementary file 1 — Supplementary Material 1 [file 10020_2024_808_MOESM1_ESM.docx]

# Authors’ responses to the editor and reviewers

**Title:** Endoplasmic reticulum stress and therapeutic strategies in metabolic, neurodegenerative diseases and cancer

**Manuscript Number:** MOME-D-23-01536

**Initial Date Submitted:** 15 Dec 2023

**Corresponding author:** Dr. Ling Ma, Department of Clinical Laboratory, Union Hospital, Tongji Medical College, Huazhong University of Science and Technology, Wuhan 430022, China. Email: malingxh@163.com

Dear Editor:

We sincerely appreciate all the efforts and suggestions devoted by the reviewers and editors to strengthen the manuscript. To address the concerns of reviewers and editors, we have revised our manuscript as per suggestions and comments. Additionally, manuscript with revised portions marking in red is also uploaded.

We hope that we have addressed the concerns raised by the reviewers and our review is now meet the requirement for publication in *Molecular Medicine*. We believe that this latest review on the mechanisms of endoplasmic reticulum stress and therapeutic targets in metabolic diseases, neurodegenerative disorders, and cancer can provide us with more effective clinical treatment strategies. If you have any additional concerns, please feel free to contact with us.

Your kind consideration is highly appreciated.

Sincerely yours,

Professor Ling Ma.

Department of Clinical Laboratory, Union Hospital, Tongji Hospital, Tongji Medical College

Huazhong University of Science and Technology

1277 Jiefang Avenue, Wuhan, Hubei 430022, China; Email: malingxh@163.com
